# Prevalence of Urinary Tract Injuries in Patients With Rectal Cancer Undergoing Low Anterior Resection Versus Abdominal Abdominoperineal Resection at a Tertiary Care Center in Latin America

**DOI:** 10.7759/cureus.93039

**Published:** 2025-09-23

**Authors:** Jesus A Sanson-Riofrio, Yamil Omar Ochoa Checa, Alain Michel Alvarado Padilla, Victor Alonso Valdez Gonzalez, Ashley Daniel Zarate Jimenez, Benjamin Ortiz Padilla, Sandra Saraí Grano Aguirre, Linadi Veronica Vallejo Silva, Fernando Miguel Ochoa Checa, Ismael Brito Toledo

**Affiliations:** 1 Colorectal Surgery, Instituto Mexicano Del Seguro Social, Centro Médico Nacional, Siglo XXI Hospital De Oncología, Mexico City, MEX; 2 General Surgery, Instituto Mexicano Del Seguro Social, Zapopan, MEX; 3 General Surgery, Instituto Mexicano del Seguro Social, Mezcales, MEX; 4 Surgery, Instituto Mexicano del Seguro Social, Tepic, MEX; 5 General Surgery, Instituto Mexicano del Seguro Social (IMSS) Hospital General #33, Puerto Vallarta, MEX; 6 General Surgery, Instituto Mexicano del Seguro Social, Puerto Vallarta, MEX; 7 General Surgery, Universidad de Guadalajara (UDG), Guadalajara, MEX; 8 General Medicine, Instituto Mexicano del Seguro Social, Tepic, MEX; 9 Medicine, Universidad Lamar, Guadalajara, MEX; 10 Colorectal Surgery, Hospital Oncología, Mexico City, MEX

**Keywords:** abdominoperineal resection, colorectal cancer, low anterior resection, prevalence, urinary tract injury

## Abstract

Introduction: Urinary tract injuries are uncommon complications in rectal cancer. However, there are no epidemiological data documenting their frequency in the patient population treated at the colorectal tumor service of the Oncology Hospital at CMN Siglo XIX.

Objective: The objective of this study is to compare and report the prevalence of urinary tract injuries in patients with rectal cancer undergoing low anterior resection (LAR) or abdominoperineal resection (APR) at a high-specialty center in Latin America.

Materials and methods: An observational, retrospective, and cross-sectional study was conducted between 2017 and 2018. Patients with histopathologically confirmed diagnoses were included, and clinical and surgical variables were recorded, including the presence of a urinary tract injury. Statistical tests for normality, bivariate analysis, and likelihood ratios were applied, considering p < 0.05 as significant.

Results: Eight cases of urinary tract injuries were identified, corresponding to a prevalence of 5.2 percent, which is higher than that reported in international literature. No injury was recorded in 94.8 percent of cases.

Conclusion: These findings suggest the need to improve intraoperative monitoring and prevention in such procedures. One of the ideal methods for ureteral identification that has recently gained popularity is indocyanine green, which is used to highlight ureteral anatomy and can serve as an alternative to methylene blue. The main advantage of this method is its accessibility, as it can be applied intraoperatively without significantly prolonging surgical time.

## Introduction

Rectal cancer represents a third of the neoplasms on a worldwide level among men and women. In Mexico, it corresponds approximately to 3 of the annual diagnoses of cancer [1]. Their principal treatment in clinic stages II and III is surgery, such as the lower anterior resection and abdominoperineal resection (APR). Besides, radiotherapy is used, which can cause adhesions and increase the risk of urinary tract injuries. This procedure implies risk of complications like damage to the ureter, bladder, or urethra. This represents a great impact on the quality of life of the patient and also on the cost of the healthcare center [2].

The prevalence of ureteral injuries in colorectal surgery is an uncommon but significant complication. Studies indicate that the prevalence ranges between 0.3% and 1.5% of the procedures performed. For example, a literature review found that ureteral injuries occur within this range during colorectal surgeries [2]. Colorectal surgical procedures correspond to the number between 5 and 15 of ureteral iatrogenic injuries [3]. Among the interventions with higher risk, lower anterior resection and abdominoperineal resection have been clearly related to a higher incidence of ureteral injuries during colorectal surgery [4]. Ureteral stenting as a prophylactic measure may reduce the risk of injury, although it is not without complications such as hematuria, urinary tract infections, and ureteral injury; furthermore, a significant reduction in the prevalence of ureteral wounds has not been demonstrated [5]. Omitting a ureteral injury is associated with increased morbidity, prolonged hospital stay, and late urological consequences in approximately one-third of patients [3]. International literature reports a prevalence of ureteral injury of 0.28 in colorectal surgery, rising to 0.7 in cases of rectal cancer and one in laparoscopic approaches [6,7]. In patients undergoing low anterior resection (LAR), the rate can reach 0.76 (7.6/1000 surgeries) [8].

At the national level, specific epidemiological data on the frequency of this event are lacking in high complexity hospitals, such as the CMN Siglo XXI Oncology Hospital. This makes it difficult to develop prophylactic protocols for the design of prevention and early detection strategies. The main objective of this observational study is to determine the prevalence of urinary tract injuries in patients with rectal cancer postoperatively with lower anterior resection or abdominoperineal resection at the CMN Siglo XXI Oncology Hospital during 2017-2018 and to identify possible associated variables such as age, gender, type of 47 surgery, pathological stage and history of radiotherapy.

## Materials and methods

A retrospective, cross-sectional, observational study was conducted in the Department of Colon and Rectal Tumors of the Oncology Hospital CMN Siglo XXI, IMSS, Mexico City, between January 1, 2017, and December 31, 2018. The records of eligible patients over 18 years of age, of either sex, diagnosed with rectal cancer, undergoing open surgery with techniques such as lower anterior resection or abdominoperineal resection were included. Urinary tract injuries were identified primarily as intraoperative findings, and in fewer cases, through the clinical manifestations presented by patients following the surgical procedure.

Exclusion criteria

Patients who did not present within the period from January 1, 2017, to December 31, 2018, were excluded.

Inclusion criteria

Patients over 18 years of age, of either sex, who underwent LAR or APR, with a confirmed diagnosis of rectal cancer. Demographic: age (years), sex. Clinical: history of radiotherapy (yes/no). Surgical: type of surgery (LAR/APR), clinical-pathological stage (0, I, II, III, IV according to TNM), T classification, presence of urinary tract lesion (defined as transection, ligation, reclassification, compression, or devascularization of the urinary tract). The 154 patients who met the criteria during the indicated period were included.

Statistical analysis

Quantitative data are presented as medians and inter-quartile ranges. Normality was assessed using the Kolmogorov-Smirnov test. Categorical variables were analyzed using the chi-square test; for nonparametric variables, the Mann-Whitney U test was used. Statistical significance was defined as p0.05. Risk was estimated using the likelihood ratio. Analyses were performed using IBM SPSS Statistics for Windows, Version 30 (Released 2024; IBM Corp., Armonk, New York, United States).

## Results

During the period from January 1, 2017, to December 31, 2018, a total of 154 patients with a histopathologically confirmed diagnosis of rectal adenocarcinoma were included. They underwent surgery with LAR or APR at the Oncology Hospital of the Siglo XXI National Medical Center of the Mexican Social Security Institute (IMSS), Mexico City.

The median age of the patients was 65 years, with an interquartile range of 58 to 72 years. Regarding sex, 90 patients (58.4%) were men and 64 (41.6%) were women. Of these, 137 patients (89.0%) received radiotherapy, while 17 (11.0%) did not.

**Figure 1:**
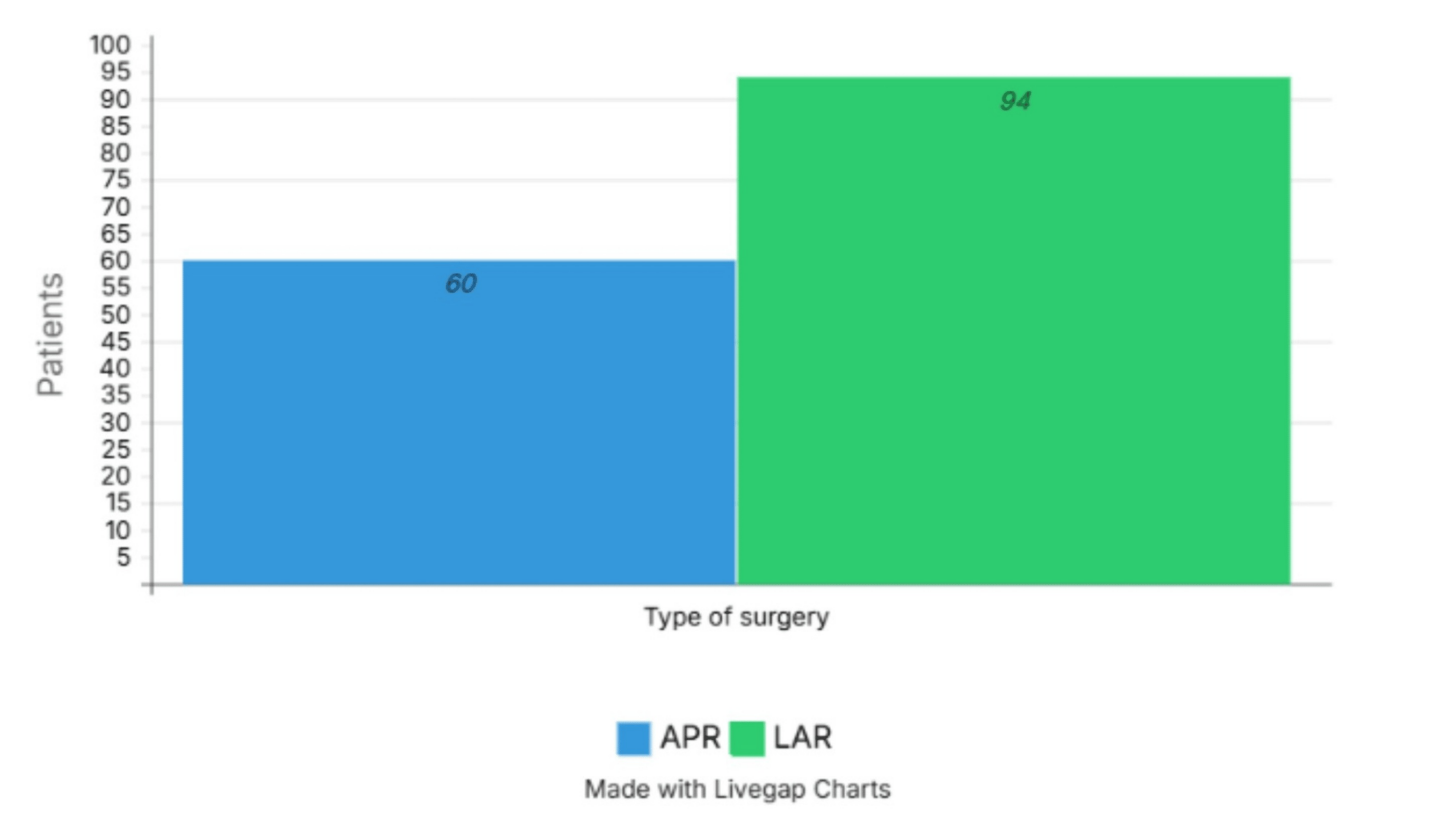
Type of surgery LAR: Low Anterior Resection; APR: Abdominoperineal Resection

**Figure 2:**
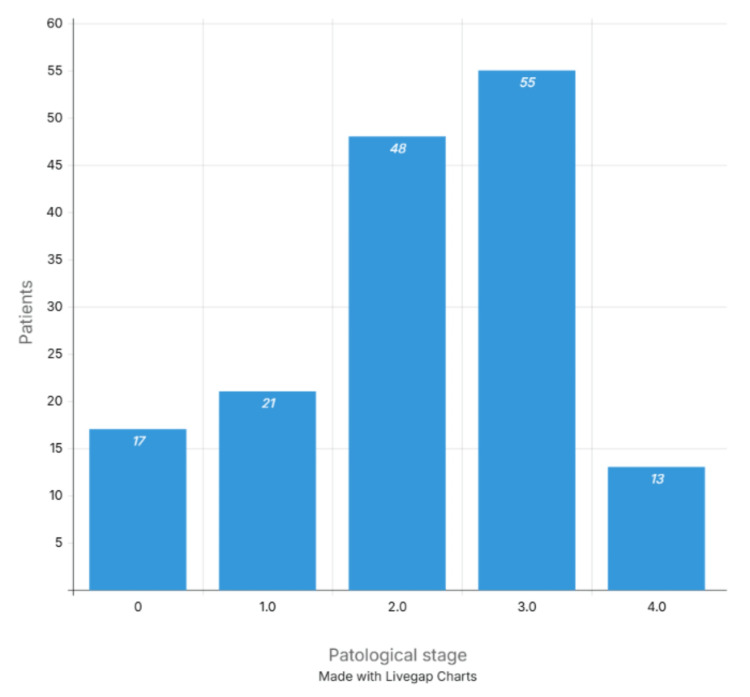
Pathological stage

The most commonly used surgical procedure was LAR in 94 cases (61.0%), while APR was performed in 60 patients (39.0%) (Figure 1). The distribution by pathological stage was as follows: stage 0, 17 cases (11.0%); stage I, 21 cases (13.6%); stage II, 48 cases (31.2%); stage III, 55 cases (35.7%); and stage IV, 13 cases (8.4%) (Figure 2).

**Figure 3:**
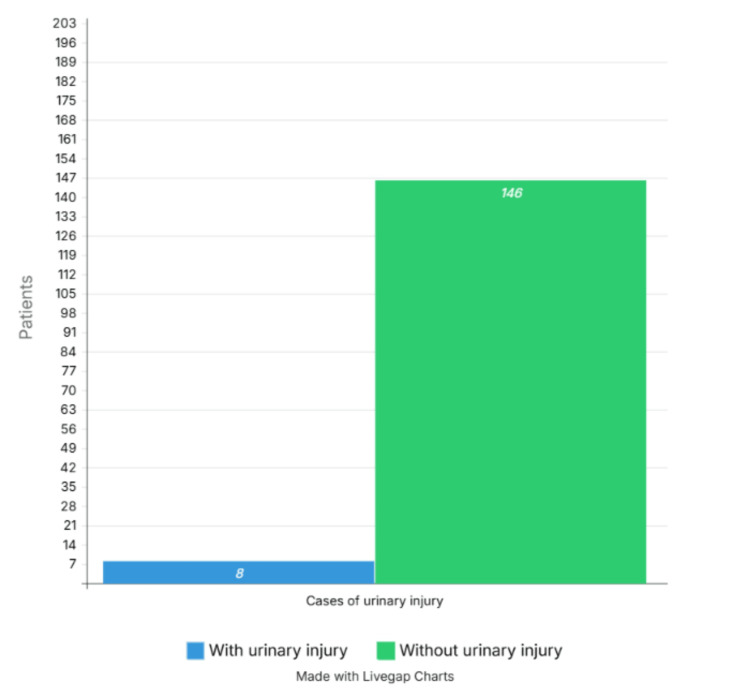
Cases of urinary injury

Eight cases of urinary tract injuries were documented, representing a prevalence of 5.2%. In the remaining 146 cases (94.8%), no urinary tract injuries were reported associated with the surgical procedure; we consider that this data is secondary to the surgical technique employed in the procedures performed (Figure 3).

Bivariate analysis

In the comparative statistical analysis between groups with and without urinary injury, no significant differences were found due to the small number of events. This is attributable to the limited sample size, as a larger cohort was not available. No statistically significant associations were found for the following variables: (i) Age: The median was 65 years in both groups. The Mann-Whitney U test revealed p = 0.802, showing no statistically significant difference; (ii) Sex: There were five cases (5.5%) of urinary injury in men and three cases (4.7%) in women, with no significant differences between the two groups (p = 0.057); (iii) Radiotherapy: Among patients who received radiotherapy, six cases (4.3%) presented urinary injury. In the group that did not receive radiotherapy, two cases (11.8%) were documented, with no statistically significant difference (p = 1.675; chi-square test); (iv) Type of surgery: In the LAR group, seven cases (7.4%) of urinary injury were reported, while only one case (1.6%) was reported in the APR group, with no statistically significant difference (p = 2.48; chi-square test); (v) Clinical-pathological stage: Two cases of urinary injury were identified in each of stages 0, I, II, and III, while no injuries were reported in stage IV. The chi-square test showed no statistical significance (p = 3.35); (vi) T classification: Injuries were documented in the following subgroups: Tis (2 cases; 11.1%), T1 (1 case; 20%), T2 (1 case; 3.2%), T3 (3 cases; 3.5%), and T4 (1 case; 6.25%). No significant association was observed (p = 4.207; chi-square test) (Table 1).

**Table 1:** Association between clinical variables and urinary injury Statistical test used: Age: Mann-Whitney U, sex, radiotherapy, type of surgery: x², pathological stage, tumor size: Kruskal-Wallis H test

	With urinary injury (n=8)	Without urinary injury (n=146)		
Variable			Total	P
Age	65	65		0.802
Sex				0.057
Female	3 (4.7)	61 (95.3)	63 (100)	
Male	5 (5.5)	85 (94.5)	90 (100)	
Radiotherapy				1.675
Yes	6 (4.3)	131 (95.7)	137 (100)	
No	2 (11)	15 (89)	17 (100)	
Type of surgery				2.48
Abdominoperineal	1 (1.6)	59 (98.4)	60 (100)	
Low anterior	7 (7.4)	87 (92.6)	94 (100)	
Pathological stage				3.35
Stage 0	2 (11)	15 (89)	17 (100)	
Stage I	2 (9.5)	19 (91.5)	21 (100)	
Stage II	2 (4.1)	46 (95.9)	48 (100)	
Stage III	2 (3.6)	53 (96.4)	55 (100)	
Stage IV	0	13 (100)	13 (100)	
Tumor size				4.207
Tis	2 (11.1)	16 (88.9)	18 (100)	
T1	1 (20)	4 (80)	5 (100)	
T2	1 (3.2)	30 (96.8)	31 (100)	
T3	3 (3.5)	81 (96.5)	84 (100)	
T4	1 (6.25)	15 (93.75)	16 (100)	

## Discussion

Colorectal cancer (CRC) is a malignant neoplasm of multifactorial origin, resulting from the abnormal proliferation and dedifferentiation of epithelial cells in the mucosa of the colon and rectum. It represents a global public health issue due to its high prevalence, significant mortality, and burden on healthcare systems [1]. A direct relationship has been observed with genetic, environmental, and dietary factors, as well as advanced age, with more than 90% of cases occurring in patients over 50 years old, as seen in our patient sample, in which the mean age was 65 years (63.6-64 years) [2].

From an epidemiological perspective, CRC is the third most common malignant neoplasm in men and the second in women worldwide [1]. In the United States, more than 134,000 new cases are reported annually, of which approximately 95,000 are colon cancer and the rest are rectal cancer [3]. In our study, men accounted for a greater number of cases than women.

The estimated incidence in Mexico in 2022 was approximately 16,000 new cases of CRC, representing around 7% of all diagnosed cancer cases in the country. The mortality in 2020 was more than 7,000 deaths from CRC, making it the second leading cause of cancer-related death in the country, second only to breast cancer [9].

Anatomically, the colon extends from the ileocecal valve to the rectum, forming a frame-like course through the abdominal cavity. It is divided into five segments: cecum, ascending colon, transverse colon, descending colon, and sigmoid colon. Each of these segments has topographical relationships with vascular, nervous, urological, and gynecological structures, which are clinically relevant for surgical approaches [5,6]. Colonic blood supply is divided between the superior and inferior mesenteric arteries, while rectal venous drainage flows into both the portal and systemic systems, explaining the potential for hepatic or pulmonary metastases depending on the tumor location [7].

Surgical treatment of rectal cancer is based on procedures designed to achieve complete tumor resection while preserving anorectal function as much as possible [8]. The most commonly used techniques are APR, LAR, and total mesorectal excision [10-12]. In our series, the most frequent type of surgery was APR, performed in 94 cases.

Injuries to the urinary tract during rectal cancer surgery, although uncommon, represent a serious complication with significant repercussions for both patients and healthcare systems [13-15]. Most urinary tract injuries occur during the ligation of mesenteric vessels, dissection at the sacral promontory, where the ureters cross the iliac artery, and in the most cephalic portion of the perineal dissection [16]. These injuries are more frequent during proctectomy and sigmoidectomy. In particular, rectal cancer resections via retro-APR have been associated with ureteral injury rates as high as 0.76 in an analysis of previous reports of ureteral trauma [17,18].

Diagnostic strategies may include direct inspection of the retroperitoneum, cystourethroscopy with assessment of ureteral flow, placement of a ureteral guidewire or catheter with or without fluoroscopy, and intravenous administration of methylene blue or indigo carmine to detect urinary extravasation [15,16]. Delayed-phase CT urography is considered the diagnostic study of choice in stable patients, according to the American Urological Association (AUA) guidelines on urotrauma [19]. Ureteral injuries may be identified by the absence of ureteral opacification, delayed or asymmetric ipsilateral nephrogram, contrast extravasation, unilateral hydronephrosis, absence of contrast in the distal ureter, presence of periureteral urinoma, or asymmetric contrast excretion with poor unilateral excretion [20,21].

Retrograde urography remains the most sensitive radiologic study to diagnose ureteral injuries, as it allows identification of the site and degree of extravasation; however, its use in acute trauma is limited due to its complexity and duration and is typically reserved for cases with persistent suspicion and inconclusive urograms [22,23]. Antegrade urography can be useful when retrograde catheter placement is not feasible, such as in complete transections with significant separation between ureteral ends; in such cases, placement of an antegrade stent or percutaneous nephrostomy is recommended [24,25].

No statistically significant risk factors were identified: age, sex, radiotherapy, type of surgery, pathological stage, or T classification showed no association with urinary injury [24,25]. Although some reports include laparoscopic surgery and advanced tumor stages as associated factors [13,18,21], in this study, the lack of association may be due to the limited number of events (n = 8) and sample size, which reduces statistical power.

Among prophylactic techniques, the use of illuminated ureteral catheters has been shown to significantly reduce the incidence of ureteral complications [24,25]. CRC in Mexico reports that in 2020, there were 14,901 new cases of CRC and approximately 6,245 deaths in the same year [26].

In Japan, a multicenter study was conducted to assess the utility of fluorescent ureteral catheters in minimally invasive colorectal surgery [27]. The results indicated that the use of these catheters can improve ureteral identification and reduce the risk of injury during surgery.

An analysis of data from the U.S. National Surgical Quality Improvement Program (NSQIP) identified 5,836 cases of iatrogenic ureteral injury among 566,036 patients undergoing colorectal surgery, representing an incidence of 1.0% [28]. Various preoperative risk factors were associated with these injuries, such as disseminated cancer and diverticular disease. These multicenter studies offer a comprehensive view of the prevalence, risk factors, and prevention strategies for ureteral injuries in colorectal surgery. The implementation of ureteral identification techniques, such as the use of fluorescent catheters, is recommended to improve patient safety during these procedures.

Early identification of ureteral injury during colorectal surgery is crucial to prevent serious postoperative complications such as infections, urinary fistulas, and irreversible kidney damage. Intraoperative detection allows immediate repair, reducing morbidity and improving long-term outcomes.

One of the most effective strategies is the use of fluorescent ureteral catheters, which utilize dyes such as indocyanine green to highlight the ureter under near-infrared light. This technique has proven to be safe and effective, allowing precise identification even in complex laparoscopic procedures. Recent studies have reported ureter identification rates exceeding 90% using this method [29].

Limitations

This study includes a relatively small number of observations, which prevents a robust multivariate analysis and limits its external validity. Additionally, the lack of a comparative control group (other centers or techniques) hinders the generalization of the results.

## Conclusions

The prevalence of urinary tract injuries in patients undergoing rectal cancer surgery was 5.19% (8/154), higher than that reported in the international literature. No statistically significant risk factors were identified (age, sex, radiotherapy, type of surgery, pathological stage, T classification). Early detection is essential: injuries identified intraoperatively required fewer interventions than those diagnosed postoperatively. It is recommended to replicate this study in a larger cohort and to consider the inclusion of active prevention techniques, such as illuminated ureteral catheters, as well as multicenter evaluations to improve external validity and multivariable analysis capacity.
